# Demographic risk factors for classical and atypical scrapie in Great Britain

**DOI:** 10.1099/vir.0.83225-0

**Published:** 2007-12

**Authors:** Darren M. Green, Victor J. del Rio Vilas, Colin P. D. Birch, Jethro Johnson, Istvan Z. Kiss, Noel D. McCarthy, Rowland R. Kao

**Affiliations:** 1 Department of Zoology, University of Oxford, South Parks Road, Oxford OX1 3PS, UK; 2 Veterinary Laboratories Agency, New Haw, Addlestone, Surrey KT15 3NB, UK; 3 School of Biological Sciences, The University of Auckland, Private Bag 92019, Auckland, New Zealand

## Abstract

Following the bovine spongiform encephalopathy (BSE) crisis, the European Union has introduced policies for eradicating transmissible spongiform encephalopathies (TSEs), including scrapie, from large ruminants. However, recent European Union surveillance has identified a novel prion disease, ‘atypical’ scrapie, substantially different from classical scrapie. It is unknown whether atypical scrapie is naturally transmissible or zoonotic, like BSE. Furthermore, cases have occurred in scrapie-resistant genotypes that are targets for selection in legislated selective breeding programmes. Here, the first epidemiological study of British cases of atypical scrapie is described, focusing on the demographics and trading patterns of farms and using databases of recorded livestock movements. Triplet comparisons found that farms with atypical scrapie stock more sheep than those of the general, non-affected population. They also move larger numbers of animals than control farms, but similar numbers to farms reporting classical scrapie. Whilst there is weak evidence of association through sheep trading of farms reporting classical scrapie, atypical scrapie shows no such evidence, being well-distributed across regions of Great Britain and through the sheep-trading network. Thus, although cases are few in number so far, our study suggests that, should natural transmission of atypical scrapie be occurring at all, it is doing so slowly.

## INTRODUCTION

For several centuries, the national sheep flock in Great Britain (GB) has been subject to infection by the fatal neurological disease scrapie (Parry, 1983). This disease was the first recognized transmissible spongiform encephalopathy (TSE), its infectious agent being an abnormal form of a prion protein. Scrapie may be linked to the occurrence of other TSEs, such as bovine spongiform encephalopathy (BSE) in cattle (Baylis *et al.*, 2002; http://www.defra.gov.uk/animalh/bse/publications/bseorigin.pdf), in turn identified as the origin of variant Creutzfeldt–Jakob disease in humans (Bruce *et al.*, 1997; Hill *et al.*, 1997). Following the transmission of BSE to humans, scrapie eradication has become a priority in both GB and elsewhere, in particular due to continuing, although declining, worries that scrapie might mask an epidemic of BSE in sheep (Kao *et al.*, 2002). In the European Union, breeding programmes have been put in place to promote scrapie-resistant genotypes, although there are concerns over the susceptibility of these genotypes to BSE (Houston *et al.*, 2003; Kao *et al.*, 2003). In the UK, this is implemented as the National Scrapie Plan (http://www.defra.gov.uk/animalh/bse/othertses/scrapie/nsp/index.html). First discovered in Norway in 1998 (Benestad *et al.*, 2003), from 2002 to date, approximately 160 UK cases of a distinct prion disease of sheep called ‘atypical’ scrapie (Everest *et al.*, 2006; Saunders *et al.*, 2006) have been detected through passive surveillance (sheep reported with clinical signs) and through two surveys: an abattoir survey (Elliott *et al.*, 2005), covering abattoirs slaughtering 94 % of UK adult sheep (http://www.defra.gov.uk/animalh/bse/publications/reports/SheepSurvey2.pdf), and a fallen stock survey (del Rio Vilas *et al.*, 2005). Worryingly, atypical scrapie cases have included sheep of genotype ARR/ARR, which are known to be resistant to classical scrapie (Saunders *et al.*, 2006). Atypical scrapie is a previously unknown prion disease, distinct from both classical scrapie and BSE (Le Dur *et al.*, 2005). Its appearance has led to continued concerns over human health and emphasizes the need for continued surveillance and scrapie eradication.

Whilst many flocks are exposed to classical scrapie via the purchase of infected sheep, only a subset go on to harbour long-term, persistent, within-flock epidemics. Studies in Ireland (Healy *et al.*, 2004) and in GB in 1998 (Hoinville *et al.*, 1999; McLean *et al.*, 1999) and 2002 (Hagenaars *et al.*, 2005; Sivam *et al.*, 2006) identified risk factors for occurrence of scrapie in the national flocks. These included large numbers of stock and greater numbers of temporary movements (e.g. overwintering and summer grazing), with additional geographical variation (McLean *et al.*, 1999; Sivam *et al.*, 2006). The risk of lambing in pens (McLean *et al.*, 1999) and high levels of infectivity in placenta suggest that perinatal transmission may be important (Touzeau *et al.*, 2006), corroborating evidence that purchase of infected ewes is a significant risk for exposure to classical scrapie (McLean *et al.*, 1999).

Scrapie transmission and control models have considered the importance of sheep movements for between-herd transmission of classical scrapie (Kao *et al.*, 2001; Gravenor *et al.*, 2001; Gubbins, 2005), but whilst mixing patterns between flocks are important, there have until recently been little data to characterize it. With the advent of the Animal Movements Licensing System (AMLS) and Scottish Animal Movement System (SAMS), the movement patterns of large livestock, including ovines, within GB are now exceptionally well-recorded. Movement data can be matched with both scrapie disease data and data from the June Agricultural Survey (JAS; http://www.defra.gov.uk/esg/work_htm/publications/cs/farmstats_web/default.htm) for individual holdings.

Recent work has linked the structure of the sheep-trading network in GB to epidemic models (Kiss *et al.*, 2006; Kao *et al.*, 2006; Green *et al.*, 2006). Here, we identify farm characteristics in terms of movement data that could be associated with incidence of scrapie, and could therefore aid in surveillance and prevalence estimation. We compare the characteristics and trading activity of atypical scrapie farms with that of classical-scrapie-notifying farms, in particular looking for evidence of associations consistent with atypical scrapie being transmissible in natural conditions.

## METHODS

### Data sources.

Our analysis concerns sheep movement data provided by AMLS and SAMS, as documented in the supplementary methods available in JGV Online, complemented by data from the JAS. The Scrapie Notifications Database (SND) identified 198 farms with cases of classical scrapie for 2004–2005. As classical scrapie diagnosis will disrupt movement, these records are compared with earlier movement data from 2003. This is not of concern for atypical scrapie, of which there were 97 records from 2002 to 2005, detected primarily by active surveillance at the abattoir. These were traced to their premises in 75 cases, to 76 different premises (see Supplementary Table S2, available in JGV Online). Data were incomplete for Shetland; therefore, the analysis below does not include this area for comparison with atypical scrapie.

### Case matching.

The association of geographical location with likelihood of a farm contracting scrapie has been documented (Hoinville *et al.*, 1999; McLean *et al.*, 1999). In our study, the effect of geographical location was removed by performing matched analyses. Each of the 198 classical scrapie farms was matched with a control farm (198 matched pairs) and each of the 76 atypical scrapie farms was matched to both a classical scrapie farm and a control farm (76 matched triplets). Triplet and pair establishment is described in the Supplementary Methods, available in JGV Online. Comparisons were made amongst pairs and triplets to determine how risk of scrapie infection is dependent upon the premises and movement variables shown in Table 1. Given the low incidence of atypical scrapie, it cannot be guaranteed that non-reporting control farms are scrapie-free in all cases. As data were markedly non-normal, a non-parametric sign test was used to test for differences between farm types. Where *a* and *b* are the numbers of positive and negative differences amongst pairs, under the null hypothesis that numbers of positive and negative differences are equal, the distribution of *a* is binomial, with parameters *p*=0.5 and *n*=*a*+*b*.

For variables with a significant association, conditional logistic regression (Stata 7.0 for Windows; Stata Corporation) was used to model the relationship between demography and scrapie risk. Only case–control pairs with complete data across all included variables were eligible for inclusion into the models. One outlier atypical farm (and thus pair), with trading activity orders of magnitude higher than all others, was excluded from the model.

### Associations between farms via sheep movements.

Numbers of direct farm-to-farm connections and indirect connections via markets were determined for the 2003 movement data. *χ*^2^ tests (or Fisher's exact test where data were sparse; Sokal & Rohlf, 1995) were used to determine whether mixing patterns of connections differed from random. For direct movements between scrapie and non-scrapie farms, numbers of movements were too small to allow use of the matched datasets, and all classical scrapie farms from 2004 to 2005 were used. For indirect connections, all movements from and to markets involving farms in the matched pairs or triplets were identified. Without identification of individual sheep or batches, farm-to-market and market-to-farm trading movements cannot be paired to give specific farm-to-farm connections. Current recommendations are that livestock remain on a market for no more than 48 h. Thus, where a movement off market occurred within 2 days of a movement onto the same market, a possible connection was assumed between the source and destination farms. Assuming no transmission at markets, only already-infected sheep pose a risk of onward transmission. Nevertheless, because we cannot identify the final locations of individual sheep, we must consider all outward movements as being potentially infectious.

The numbers of expected movements between end points of each type (nine combinations) were calculated, assuming completely random, proportionate mixing, and Fisher's exact test (expected cell counts being too small to allow *χ*^2^ tests) was used to determine whether the observed movement patterns differed from the expectation. Proportionate mixing assumes that the strength of contact between two farms is directly proportional to the product of their two contact rates, with no assortativity of mixing between particular farm types.

### Movement-network communities.

In addition to classifying farms according to region, we also classify farms according to ‘community’. Members of a community trade sheep amongst themselves more often than between communities and may be of geographical nature, or represent sectors of an industry. This presents a more natural grouping of the stratified sheep industry than is provided by geographical analysis. Communities were identified based on 2003 movement data by using the ‘Q algorithm’ (Newman, 2004), modified as described previously (Kao *et al.*, 2006), considering the movements as a static, undirected network over this period, with network connections weighted doubly where movements in both directions exist. Distributions of premises affected by atypical scrapie across regions and communities were investigated by using *χ*^2^ tests.

For classical scrapie, Kao *et al.* (2007) divided incidence into a matrix of communities and regions and tested for differences in incidence between ‘core’ elements (the matrix element with the largest number of farms for each column or row) versus the ‘fringe’ elements (the rest of the column or row). We perform a similar analysis here for atypical scrapie, assuming numbers of atypical scrapie farms to be distributed binomially amongst the sampled farms.

## RESULTS

### Matched-pairs analysis

Matched-pairs analysis using the sign test identified variables differing significantly between classical scrapie and control farms and between atypical scrapie and control farms (Tables 1 and 2). Pairs where one farm had missing JAS flock size or no movements were excluded from the corresponding test. Flock size was statistically significantly higher in scrapie farms of both types (classical scrapie, *P*<0.001; atypical scrapie, *P*=0.040). The number of off movements for scrapie farms of both types (classical, *P*=0.004; atypical, *P*=0.006) and of on movements for classical scrapie farms alone (*P*=0.038) were also statistically significantly higher. Also statistically significantly higher for both types of farm were numbers of sheep traded via off movements (classical, *P*<0.001; atypical, *P*=0.002), total moves in both directions (classical, *P*<0.001; atypical, *P*=0.003) and total sheep traded in both directions (classical, *P*<0.001; atypical, *P*=0.008). When comparing atypical and classical scrapie farms, no variables were significant.

Conditional logistic regression was used to explore further the relationships between the variables identified above (Table 2). The conclusions were similar for both atypical and classical scrapie: flock size was the most significant variable in determining scrapie risk. When both flock-size and movement variables were included in the model, no movement variable remained significant, showing the significance of the movement variables to be due to their correlation with flock size: larger farms tend to move more sheep. No significant interaction terms were observed.

### Movement-network analysis

In the 2003 movement data, 104 393 movements occurred directly between pairs of farms (all JAS premises except those identified definitively as not being farms). Each movement end point was identified as an atypical scrapie farm, a classical scrapie farm (2004–2005 data) or non-reporting (Table 3). The 11 classical–classical farm-to-farm movements were 3.8 times higher than expected from proportionate mixing. Although mixing between classical scrapie and all non-reporting farms differed significantly from random mixing (*P*<0.001), the difference is in practice small, with such a small number (*n*=11) of between-scrapie-farm movements compared with the number of reporting farms (*n*=198). No such departure from random mixing was obtained for atypical scrapie or non-reporting farms (*P*=1). However, the number of direct farm–farm movements involving the small number of atypical scrapie farms was small and the power of the test low. Two or more direct atypical–atypical moves would be sufficient to result in a significant departure from random mixing, but this would correspond to 8.8 times greater than expected. Zero cell counts precluded the use of the same test for mixing between classical and atypical scrapie farms.

Potential farm-to-farm trading links via markets were evaluated as described above. From the 76 matched triplets, there were 2868 off movements to markets in 2003, and 611 on movements from markets. Matching on and off movements within 2 days, there were 877 possible movements between cases and controls via markets. Excluding movements with equal sources and destinations, and multiple routes of connection, there were 356 possible connections amongst cases and controls, tabulated by source and destination farm type in Table 3. There were no significant differences amongst the three types of farms concerning how often they were a source of a possible connection (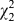
=0.34; *P*=0.84), although the corresponding test for destinations of possible connections was significant (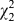
=31.4; *P*<0.001). This should not be taken as indicative of more actual movements from markets to classical scrapie farms: there was no significant difference in either number of sheep received from markets (*P*=0.20), or movements from markets (*P*=0.45) to classical and atypical scrapie farms in paired comparisons. A *χ*^2^ test showed no departure from random mixing for these connections, and thus no associativity amongst atypical or classical scrapie cases via markets (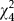
=3.54; *P*=0.47). Also, no significant associativity was noted in the larger sample of 198 classical–control pairs.

### Geographical distribution of cases

**Fig. 1. f1:**
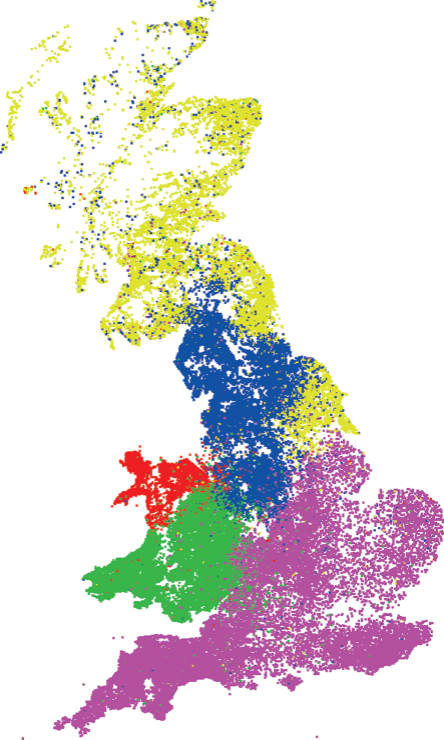
Community membership of sheep-trading farms in 2003.

Movement-network community analysis revealed five large communities of varying size, as shown in Fig. 1. These are largely geographical, but with small numbers of each community scattered over the whole country.

The 76 premises affected by atypical scrapie and 291 by classical scrapie (all 2002–2005 data) were classified according to one of six GB regions and the five communities as shown in Fig. 1 and Supplementary Fig. S1 (available in JGV Online). For ease of comparison of numbers of classical and atypical scrapie between regions or communities, Table 4 includes normalized ratios of relative incidence, calculated as
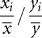
where the fraction of premises affected in region or community *i* is given as *x_i_* for atypical and *y_i_* for classical scrapie. Division by the same quantities measured across the whole study area, 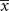 and 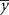, then provides normalized measures of incidence. Atypical incidence is taken as the proportion of positively sampled farms from the abattoir survey for each network community or region, and classical incidence as the proportion of total holdings affected. This controls for the effect of a suspected sampling bias of the abattoir survey, which sampled most intensively in Wales and the West Midlands (Birch *et al.*, 2006). Farms recorded on the census as holding fewer than 70 sheep were excluded when calculating total farms in a region or community, to allow fairer comparison with the 2002 Anonymous Postal Survey on Scrapie (ASS; Sivam *et al.*, 2006), which did not examine farms holding fewer than 30 breeding ewes (assuming a lambing ratio of approx. 2). Accounting for sampling bias, regional incidence of atypical scrapie *x_i_* did not differ significantly from random across all sampled premises (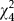
=6.1; *P*=0.19), although with relatively high detection rates in animals from Scotland and the north-east of England. Similarly, atypical scrapie distribution was not concentrated significantly in particular network communities (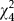
=5.9; *P*=0.21), although again with relatively high detection rates in animals from the Scotland and north-east England community (yellow in Fig. 1). Atypical incidence may alternatively be normalized according to the ASS (Sivam *et al.*, 2006), as shown for different regions in in Table 4, which also indicates a relatively higher risk of atypical scrapie in the north-east of England. Classical scrapie distributions differed significantly from random mixing across both communities and regions (*P*<0.001). Following the procedure described previously (Kao *et al.*, 2007), no significant difference in incidence of atypical scrapie was found amongst ‘core’ and ‘fringe’ areas of network communities, suggesting them to be homogeneous.

## DISCUSSION

The small number of atypical scrapie cases so far are distributed across all regions of GB and across all communities in the sheep-trading network, with no strong evidence of varying incidence. As with classical scrapie, farms with atypical scrapie tend to be larger farms dealing with more stock, and none of the variables studied distinguish between farms with the two types of scrapie.

Whilst there is evidence of higher than average association amongst farms with classical scrapie, there was none between farms affected by atypical scrapie through movements in 2003; furthermore, in the 2003 data, there are no direct movements at all between farms associated with atypical scrapie. These results are similar to, but differ slightly from, the results of Kao *et al.* (2007). This arises because the results of the present paper are based upon a larger sample of movement data. Nevertheless, the fundamental result is the same – the existence of weak interactions between farms apparent in the movement data. This assortativity between scrapie farms might be a signal of transmission between them. However, in an industry as highly structured as sheep farming in the UK, any variation in scrapie risk across sectors of the industry (e.g. breeds) might be reflected in such assortativity in the movement network, even in the absence of direct farm-to-farm transmission. The present analysis does not allow these two mechanisms to be distinguished.

The time frame of movements used in both studies is quite short compared with the typical age of affected sheep. More associations might be expected if a longer period of movements, more consistent with a long infection window, were used. Given that the mean duration of a large within-farm epidemic of scrapie has been estimated at 15 years (Gravenor *et al.*, 2001), this time frame could be considerable. Additional links between infected farms through movement of infected animals are possible through markets and, in GB, movements through markets account for the majority of links between farms (Kiss *et al.*, 2006).

AMLS and SAMS are not, however, designed to allow pairing of source and destination farms of movements through markets, which limits the utility of the data in this area. How far a single infected sheep can spread through the network, and hence the potential rate of spread of a disease with long latent period such as scrapie, cannot be determined precisely. Tracing of individuals over several years might enable determination of, for example, whether it is feasible for atypical scrapie cases to have had a common origin in GB or, if one is assumed, how long ago this might have arisen. Additionally, not all movements are of equal epidemiological importance: movements of breeding stock are of prime importance in classical scrapie transmission, but many movements are of lambs, which could obscure the epidemiological picture. The data do not allow these types of movements to be distinguished.

Analysis of scrapie-incidence data is complicated by differences in sampling and reporting rates across time and space (del Rio Vilas *et al.*, 2006). Furthermore, the cases analysed in this paper represent different sampling processes: the classical cases are from passive surveillance, whilst the atypical cases are mostly from active surveillance. Notifications of classical scrapie have been rising in Wales from 2002 to 2005, during which time notifications have fallen somewhat or remained level elsewhere. This is as likely to be due to changes in policy as real changes in the underlying incidence of disease. This is reflected by the anonymous survey data, which identify different areas with highest prevalence compared with the locations of notified classical scrapie cases. Regional variation in genotype frequencies could also cause differences of incidence in classical and atypical scrapie, resulting in regional ‘hot spots’ for one type or the other. More data are required before these potential factors may be discounted.

Farms susceptible to atypical scrapie appear to be similar to those susceptible to classical scrapie in terms of the demographic variables studied here, i.e. large farms trading many sheep. Further, there is only weak evidence for associations amongst classical scrapie-affected farms and none for atypical scrapie, albeit with fewer cases of the latter to analyse. Similar to a previous study (Lühken *et al.*, 2007), our results are consistent with atypical scrapie being, at most, weakly transmissible. The hypothesis that natural transmission of atypical scrapie occurs at most at a similar rate to classical scrapie may appear unsurprising, but is nevertheless important. Whilst it is impossible to make a direct comparison across such widely different time frames and/or species, were atypical scrapie to be an emerging TSE, rapid, widespread transmission could occur, as was the case with classical scrapie in the 1800s (Parry, 1983) or as is occurring now with chronic wasting disease in North American cervids (Williams, 2005), in both cases with devastating consequences to the host population. Given its incidence in ‘scrapie-resistant’ genotypes, atypical scrapie may yet provide a major challenge to scrapie eradication, and selective breeding schemes might in the future need to be revised. However, at present there is no indication that the scientific basis for scrapie eradication has eroded.

## Supplementary Material

[Supplementary methods]

## Figures and Tables

**Table 1. t1:** Premises variables used for sign tests (2003 data) Variables thus found to be significant were then used for conditional logistic regression.

**Variable**	**Detail**
Number of moves (batches)	Total moves, off only, on only*
Number of sheep moved	Total sheep, off only, on only*
Mean distance of animal movements	Overall, off only, on only
Flock size	

*Variables sharing information that cannot all be introduced into the same model.

**Table 2. t2:** Binary logistic regression models of scrapie risk (2004–2005 data) Ninety-five per cent confidence intervals for odds ratios are given. The best-fit models using flock size and movement data are shown.

**Model**	**Variable**	**Odds ratio**	***P***	**pseudo-*r*^2^**
Classical	Flock size	2.89 (1.91–4.38) per 1000 sheep	<0.001	0.22
Classical	Sheep moved	1.62 (1.28–2.05) per 500 sheep moved	<0.001	0.11
Atypical	Flock size	2.24 (1.13–4.42) per 1000 sheep	0.020	0.17
Atypical	Moves off	2.03 (1.33–3.11) per 10 moves	0.001	0.18

**Table 3. t3:** Observed movement patterns between farms: direct (using all premises data) and via markets (using premises in triplet analysis only)

**Destination type**	**Source type**		
	**Non-reporting**	**Classical**	**Atypical**
**Direct farm–farm movements**			
Non-reporting	103 276	416	186
Classical	377	11	0
Atypical	127	0	0
**Observed possible farm-to-farm links via markets**			
Non-reporting	29	36	35
Classical	66	50	52
Atypical	28	28	32

**Table 4. t4:** Incidence of atypical (abattoir survey data) and classical (2002–2005 data) scrapie according to geographical region (Supplementary Fig. S1) and sheep-trading communities (Fig. 1)

	**Cases (*n*)**	**Atypical incidence (‰)**	**Classical incidence (‰)**	**Normalized incidence relative to classical**	**Normalized incidence relative to anonymous survey**
**Regions**					
Scotland	10	9.1	2.7	3.65	1.11
Wales	29	5.7	12.5	0.49	1.16
South-west	5	4.9	9.6	0.54	0.54
North-west	3	8.8	5.3	1.75	1.48
North-east	6	16.3	6.6	2.62	2.81
Midlands and south-east	7	6.1	5.6	1.16	0.99
Communities*					
North Wales (red)	7	6.1	6.5	0.92	
North West (blue)	8	9.7	5.2	1.82	
South Wales (green)	24	5.2	10.6	0.48	
South and east (purple)	10	8.0	7.3	1.08	
Scotland and north-east (yellow)	10	11.5	3.0	3.77	

*Colours in parentheses refer to Fig. 1.
