# Supplementary material for: Demographic risk factors for classical and atypical scrapie in Great Britain
Source: J Gen Virol. 2007 Dec;88(Pt 12):3486–92. doi: 10.1099/vir.0.83225-0 (PMC2884981; doi:10.1099/vir.0.83225-0)
Supplement: [Supplementary methods] [file supp_88_12_3486__index.html]

 Demographic risk factors for classical and atypical scrapie in Great Britain -- Green et al. 88 (12): 3486 Data Supplement - Supplementary methods -- Journal of General Virology

### Demographic risk factors for classical and atypical scrapie in Great Britain, by D. M. Green, V. J. del Rio Vilas, C. P. D. Birch, J. Johnson, I. Z. Kiss, N. D. McCarthy and R. R. Kao

*Journal of General Virology* vol. **88**, part 12, pp. 3486 – 3492

**Supplementary Methods.** Including Supplementary Tables S1 and S2 and Supplementary Fig. S1 [PDF] (85 KB)

  
  
