# Supplementary material for: Demographic risk factors for classical and atypical scrapie in Great Britain
Source: J Gen Virol. 2007 Dec;88(Pt 12):3486–92. doi: 10.1099/vir.0.83225-0 (PMC2884981; doi:10.1099/vir.0.83225-0)
Supplement: [Supplementary methods] [file supp_88_12_3486__1.pdf]

Supplementary methods

Data sources

Our analysis concerns movement and census data for sheep provided by the June Agricultural Census (JAS), Animal Movements Licensing System (AMLS) and Scottish Animal Movement System (SAMS). Each movement record consists of a date, number of animals moved and the CPH (county/parish/holding) identifier of the source and destination premises, be they agricultural holdings, markets or other types of premises. Combining the SAMS and AMLS data resulted in a table containing 1 120 890 movement records from 2003. This time frame was chosen so as to avoid the 'teething' problems associated with the first such data, but to allow relation of scrapie incidence to earlier movement data, where the trading patterns of the scrapie farms would be presumably unaffected.

Many records were duplicates with identical date, source, destination and batch size. Such duplicates were removed, reducing the number of records to 891 989. This therefore gives a conservative estimate of the numbers of movements, as some duplicates may be real movements. In some records, either the source or the destination CPH code were missing, but these still provide useful data for the other end-point of the movement.

The JAS for 2003 provides geographical locations, premises type and reported stock held for each CPH code. Removing 798 duplicate records from the JAS gave a table containing 509 177 records (covering types of farming enterprise other than just sheep farms). Movement data and the JAS were cross-referenced and the following summary statistics were obtained for each premises: numbers of movements (on and off separately, and combined), numbers of sheep moved (on, off and combined), mean distance of movements (on, off and combined) and reported stock held. Premises were also classified into one of six large regions, based on the county code of the premises CPH (Supplementary Fig. S1).

The Scrapie Notifications Database (SND) identifies each of 2943 confirmed scrapie cases by CPH of confirmation and date, belonging to 616 premises between 1998 and 2005. The more restricted set of cases from 2002–2005 alone (coinciding with the period of data known for atypical scrapie) consisted of cases in 291 premises (excluding Shetland) and, for 2004–2005 alone, 198 premises (176 excluding Shetland). There were 97 records of atypical scrapie from 2002 to 2005, detected primarily by active surveillance (mostly at the abattoir), which were traced to their premises in 75 cases, to 76 different premises, as

Green, D. M., del Rio Vilas, V. J., Birch, C. P. D., Johnson, J., Kiss, I. Z., McCarthy, N. D. & Kao, R. R. (2007). Demographic risk factors for classical and atypical scrapie in Great Britain. *J Gen Virol* **88**, 3486–3492.

shown in Supplementary Table S2. For the analyses of relative incidence in the abattoir survey, the known natal holding was used where known (assuming preponderance of perinatal transmission; two of the 60 premises). Livestock-trading behaviour is not expected to be altered as a result of atypical scrapie detection in these data. Therefore, we use the longer time frame for atypical scrapie to maximize the numbers of cases available for analysis.

A fourth data source, the 2002 Anonymous Postal Survey on Scrapie (ASS; Sivam *et al.*, 2006), was used to compare incidence of scrapie in different data sources.

Demographic factors

Scrapie farms were paired with both control farms without scrapie notification (198 classical scrapie pairs) and, in the case of atypical scrapie farms, additionally with classical scrapie farms in the same county (76 classical/atypical/control triplets). Matches were made at random within the population of suitable matches, but four criteria determined eligibility of a match.

County. Matches were made to farms in the same county to account for regional variation in farming practices. For 14 atypical scrapie farms, mostly in counties with fewer farms, matching classical scrapie farms were only available in adjoining counties. However, this alone is not indicative of regional differences in the distribution of the two forms of scrapie.

Parish. Matches were made where possible to farms in the same parish, in addition to county.

Movement data. Matches were made preferentially to farms with sheep movement data during 2003, as the JAS also contains data on non-sheep farms and choosing only actively trading farms avoids selecting these as a match. This method may risk introducing a sampling bias, but maximizes the amount of relevant data available for analysis.

Census data. Where no match was available with movement data, matches were made preferentially to farms with sheep recorded on the JAS.

None of the atypical scrapie farms had a history of scrapie since 1998.

Supplementary Table S1. Traced case numbers of scrapie, 2002–2005

	Classical 2002–2005	Classical 2004–2005	Atypical
All	342	198	75
Excluding Shetland	291	176	75

Supplementary Table S2. Numbers of atypical scrapie farms and reported cases according to source

Source	Sheep reported	No. traced	No. traced premises
Fallen stock	10	10	11
Passive surveillance	4	4	3
Abattoir survey	83	61	62
**Total**	**97**	**75**	**76**

Reference

Sivam, S. K., Byalis, M., Gravenor, M. B. & Gubbins, S. (2006). Descriptive analysis of the results of an anonymous postal survey of the occurrence of scrapie in Great Britain in 2002. *Vet Rec* **158**, 501–506. Medline

Author contributions

This project was conceived by R. R. K. Scrapie-notification data were processed and provided by V. J. d. R. V. and C. P. D. B. and analysed by D. M. G. Some analyses were performed by I. Z. K. The analyses were conceived by J. J., N. D. M., D. M. G. and R. R. K. The manuscript was written by D. M. G. and R. R. K. All authors discussed the interpretation of results and commented on the manuscript.

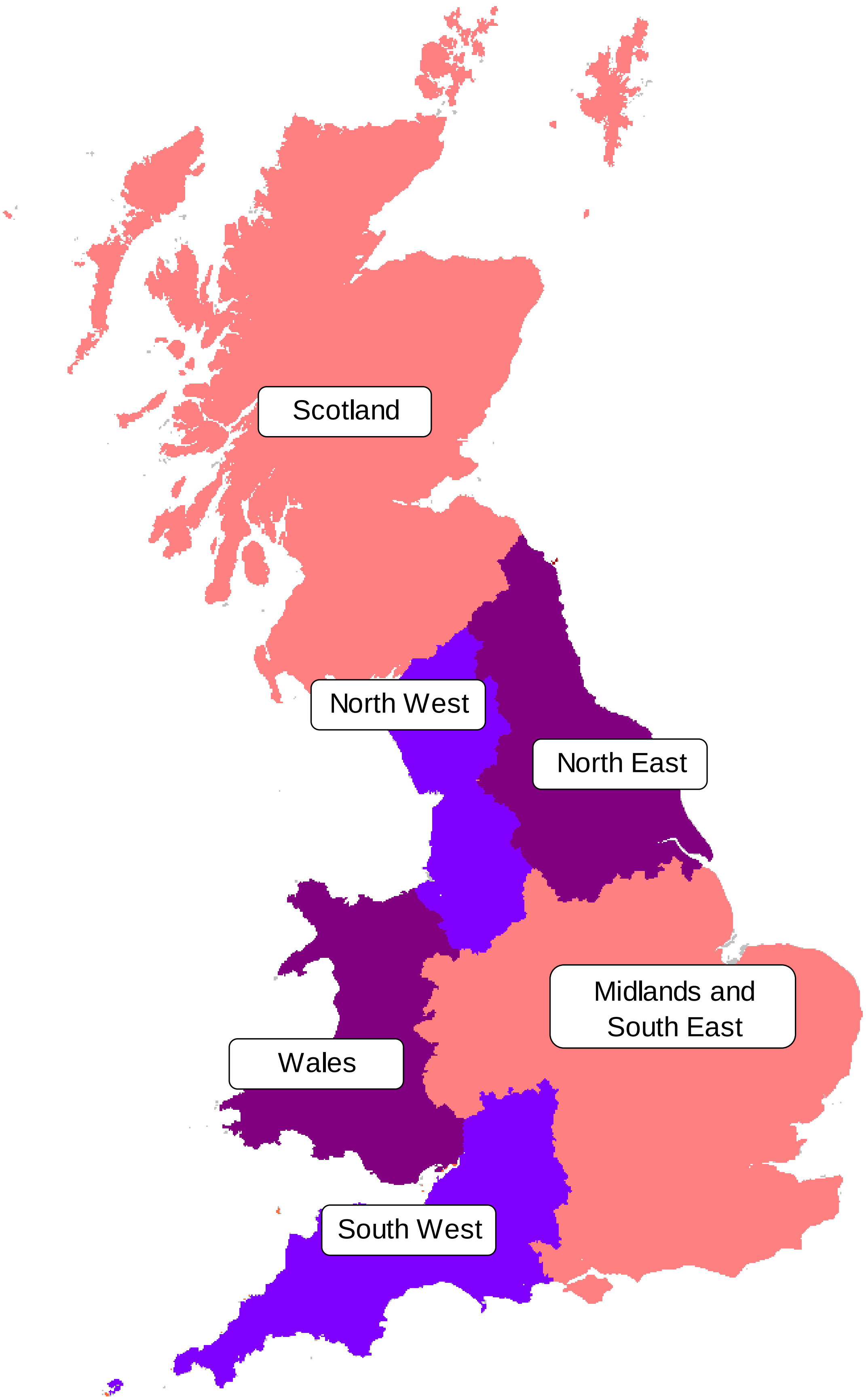

Supplementary Fig. S1. Regions of Great Briatin used in the analysis.

Green, D. M., del Rio Vilas, V. J., Birch, C. P. D., Johnson, J., Kiss, I. Z., McCarthy, N. D. & Kao, R. R. (2007). Demographic risk factors for classical and atypical scrapie in Great Britain. *J Gen Virol* **88**, 3486–3492.
